# Withdrawal of antibiotic growth promoters in China and its impact on the foodborne pathogen *Campylobacter coli* of swine origin

**DOI:** 10.3389/fmicb.2022.1004725

**Published:** 2022-09-08

**Authors:** Renqiao Wen, Chao Li, Mengyu Zhao, Hongning Wang, Yizhi Tang

**Affiliations:** ^1^Key Laboratory of Bio-Resource and Eco-Environment of Ministry of Education, College of Life Sciences, Sichuan University, Chengdu, Sichuan, China; ^2^Animal Disease Prevention and Food Safety Key Laboratory of Sichuan Province, College of Life Sciences, Sichuan University, Chengdu, Sichuan, China

**Keywords:** *Campylobacter*, antibiotic growth promoters, swine, antibiotic resistance, virulence factors (VFS)

## Abstract

Antibiotic growth promoters (AGPs) have been used as feed additives to improve feed efficiency in food animals for more than six decades. However, the wide use of AGPs has led to the emergence of antibiotic-resistant pathogens of animal origin, posing a significant threat to food safety and public health. China prohibited the addition of AGPs to animal feed from July 2020. The impacts caused by the withdrawal of AGPs on the prevalence and antibiotic resistance of foodborne pathogens have not been illustrated. Here, a total of 471 strains of *Campylobacter* were isolated from pigs from three pig farms and two slaughterhouses in Sichuan Province for 4 consecutive years (2018–2021), including 2 years before and 2 years after the ban on AGPs in China. The isolation rate of *Campylobacter* had a slight increase after prohibiting the addition of AGPs to the feed. Contrary to what we expected, the antibiotic susceptibility test and WGS data showed that the antibiotic resistance to gentamicin and florfenicol and the abundance of virulence genes increased significantly after the ban of AGPs. Comparison of the isolates of swine origin with isolates of human origin indicated the potential of antibiotic-resistant *Campylobacter* transmission from pigs to humans. These data suggested that phasing out AGPs may lead to increased use of therapeutic antimicrobials, promoting the prevalence and transmission of both antibiotic resistance and virulence genes.

## Introduction

In the initial stage of antibiotic use, most of them were shared by humans and animals for the purpose of treatment (Richou, 1952). In 1950, the United States first added antibiotics to animal feed as livestock and poultry antibiotic growth promoters (AGPs), and then this strategy was widely used in other countries, including China (Dibner and Richards, 2005). Industries and farmers have benefited from AGPs due to increased productivity. However, with the increasing research on the association of the antibiotic resistance of bacteria and the AGPs used in animals (Elliott and Barnes, 1959; Bush et al., 2011; Wang et al., 2017), recommendations to ban AGPs in animal feed have been discussed by scientists and politicians. The first nation to eliminate the use of antibiotics for growth promotion was Sweden in 1986 (Dibner and Richards, 2005), and since then, many countries have prohibited the addition of AGPs to animal feed (Maron et al., 2013; Salim et al., 2018). China also adopted this legislation, and the regulations came into force on July 2020, banning 11 antibiotics, including bacitracin zinc premix, flavomycin premix, virginiamycin premix, natriuretic peptide premix, avilamycin premix, kitasamycin premix, hygromycin calcium premix, chlortetracycline premix, enramycin premix, bacitracin methylene salicylate premix, and quinolone premix, as feed additives.

After the introduction of a ban on the use of AGPs for livestock, the selection pressure of antibiotics on bacteria was relieved, and the antibiotic resistance profile of intestinal bacteria changed rapidly (Wang et al., 2020). However, there are still many antibiotics used for the treatment of bacterial infections. According to this, we hypothesized that the prevalence and the antibiotic resistance profile may be affected by this prohibition and investigated an important member of the swine gut microbiota, *Campylobacter* spp., with a whole-genome sequencing approach. *Campylobacter* species are among the most frequently identified bacterial causes of human gastroenteritis (Kaakoush et al., 2015). As a foodborne pathogen, *Campylobacter* is constantly exposed to antimicrobial agents. To counteract selection pressure, *Campylobacter* has developed various mechanisms, including mutations in target genes, such as 23S rRNA mutations to macrolides and *gyr*A mutation to fluoroquinolones (Tang et al., 2017), multidrug efflux pump *Cme*ABC or *RE-Cme*ABC (Lin et al., 2002; Yao et al., 2016), and horizontally acquired antibiotic resistance genes, such as rRNA methylase *Erm*(B) (Wang et al., 2014). Some of the mechanisms confer resistance to a specific class of antimicrobials, while others may confer multidrug resistance. In recent years, we have also identified the cross-resistance genes *optr*A and *cfr*(C), conferring resistance to structurally unrelated antibiotics (Tang et al., 2020). Of particular concern is resistance to the oxazolidinone class, which is used as the last resort for treating MDR Gram-positive bacterial infections in humans.

The mechanism by which *Campylobacter* infects the host and induces disease is complex and unique. Unlike classical virulence factors such as T3SS and lipopolysaccharide (LPS) observed in other enteropathogens such as enterotoxigenic *E. coli* and *Salmonella*, *Campylobacter* lacks or does not extensively rely on many classical virulence factors (Gaytán et al., 2016). *Campylobacter* has a series of special virulence genes.

The classification of major virulence factors in *Campylobacter* includes motility, invasion, toxin, adhesion, effect delivery system, and immune modulation (van Vliet et al., 1999). The mobility of *Campylobacter* is encoded by coding genes, but the flagella not only acts as a motor organ but also acts as a secretion device, which can transfer invasion-related virulence factors such as the *Campylobacter* invasion antigen (Cia) protein, which is delivered into the cytoplasm of the host (Rivera-Amill et al., 2001; Guerry, 2007). Effector delivery systems include not only flagella but also the type IV secretion system, which is used to secrete effectors such as CDT (cytolethal distending toxin) (Kienesberger et al., 2011). The immunomodulatory system of *Campylobacter* mainly includes capsule and lipooligosaccharide (LOS), which play an important role in bacterial survival and persistence in the environment and in evasion of host immune responses. The presence of heptose residues in the capsule may be important for virulence (Karlyshev et al., 2005). LOS diversity is significant for the ability to colonize diverse hosts and gut niches (Guerry et al., 2000). One should pay attention to the virulence factors of *Campylobacter* because the presence and abundance of *Campylobacter* virulence genes can assess the potential threat of the strain to the host.

To analyze the evolutionary changes in *Campylobacter* after the ban of AGPs, we carried out an extensive and consecutive sampling regime in Sichuan Province for 4 years, including 2 years before the ban of AGPs and 2 years after that.

## Materials and methods

### Sample collection, *Campylobacter* isolation, and identification

*Campylobacter* strains were isolated under two conditions: (1) AGPs could be used as feed additives for livestock (in 2018 and 2019) and (2) after the ban on the addition of AGPs to the feed (after July 2020 and 2021). A total of 1,090 swine fecal samples and cecal contents were collected from three pig farms and two pig slaughterhouses in Sichuan Province, which is one of the largest pig raising provinces in China, for four consecutive years, including 2018 (*n* = 334), 2019 (*n* = 376), 2020 (*n* = 110), and 2021 (*n* = 120). Fresh swine fecal samples or cecal contents were collected and streaked onto Skirrow agar plates containing *Campylobacter* selective supplements and incubated at 42°C for 48 h under microaerobic conditions (5% O_2_, 10% CO_2_, and 85% N_2_). Two sets of previously published PCR primers were used to identify and differentiate *C. jejuni* and *C. coli* (Linton et al., 1996, 1997).

### Antimicrobial susceptibility testing

In total, 200 *C. coli* (50 from each year) isolates were randomly chosen and assayed in the antimicrobial susceptibility testing. The broth microdilution method as recommended by CLSI was used to determine the susceptibility of *Campylobacter* isolates to eight antibiotics (florfenicol, linezolid, gentamicin, tetracycline, lincomycin, erythromycin, ciprofloxacin, and ampicillin), and the antimicrobial resistance breakpoints were chosen according to the interpretive standards established by the CLSI (Clinical and Laboratory Standards Institute, 2010; Watts et al., 2013). *C. coli* 33559 was used as a quality control strain.

### Whole-genome sequencing

Genomic DNA of 120 *C. coli* isolates (30 from each year) was extracted using the Wizard Genomic DNA purification kit (Promega). The libraries were prepared using a TruSeq DNA PCR-free library preparation kit, and whole-genome sequencing was performed on an Illumina MiSeq platform (Shanghai Personal Biotechnology Co., Ltd., China) using a 350-bp paired-end library with ∼200-fold average coverage. The paired-end reads were assembled *de novo* using SOAPdenovo v2.04 and Gapcloser v1.12.

### Antimicrobial resistance genes, virulence genes, and multilocus sequence typing analysis

All contigs were searched for antimicrobial resistance genes (ARGs) by ResFinder 3.2^1^ (Florensa et al., 2022). Virulence genes (VGs) of *Campylobacter* isolates were obtained by comparing the assembled draft genome with the online website of the VFDB database^2^ (Chen et al., 2005). All the VGs were grouped according to the information from the VFDB. Heatmaps showing ARG co-occurrence and exclusivity and virulence genes were generated using the R package (ggplot2, default parameters). Multilocus sequence typing (MLST) was used to search for the sequences of seven selected *Campylobacter* housekeeping genes, *asp*A (aspartase A), *gln*A (glutamine synthase), *glt*A (citrate synthase), *gly*A (serine hydroxymethyltransferase), *pgm* (phosphoglucomutase), *tkt* (transketolase), and *unc*A (ATP synthase alpha subunit), using the online website MLST 2.0^3^ (Larsen et al., 2012).

### Phylogenetic analysis and pan-genomic analysis

A total of 225 genomes, including 120 of this study and 105 *C. coli* isolates of patient origin downloaded from the National Center for Biotechnology Information (NCBI) database, were used to generate a core-genome single nucleotide polymorphism (SNP) alignment and construct a phylogenetic tree using CSI Phylogeny 1.4^4^ (Kaas et al., 2014). The phylogenetic tree was annotated in iTOL^5^ (Letunic and Bork, 2021). The fasta sequences of 120 strains of swine-derived *Campylobacter coli* were annotated by RAST^6^ to obtain genbank files (Aziz et al., 2008). Pan-genomic analysis of these genbank files was performed by GView Server^7^ (Petkau et al., 2010) using the default parameters: percent identity cutoff (90), alignment length cutoff (100), and *e*-values (<1e−10).

### Statistical analysis

Statistical analysis was performed on the data using the software SPSS, two-sided test, and the data with *P* < 0.05 were considered significantly different.

### Data availability

All data analyzed in this study are publicly available. The whole-genome sequencing data downloaded from NCBI are listed in Supplementary Table 1. The genomes of the 120 newly sequenced *C. coli* isolates were deposited in the NCBI database under accession number PRJNA817390.

## Results

### Prevalence of *Campylobacter* before and after antibiotic bans in fodder

**FIGURE 1:**
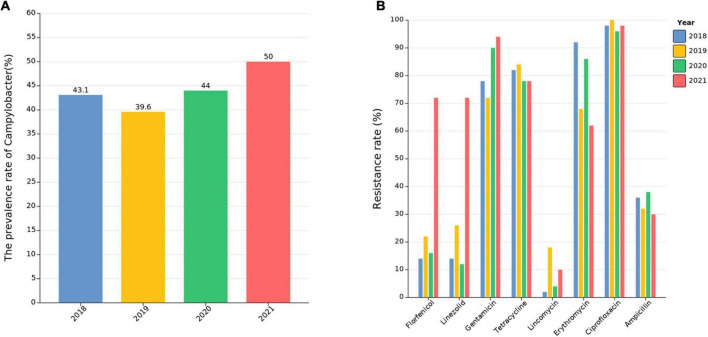
The prevalence and antibiotic resistance rate in *Campylobacter* isolates. **(A)** The prevalence rate of *Campylobacter* over 4 years. **(B)** Antibiotic resistance rates of isolated *Campylobacter* spp. as determined by antibiotic susceptibility testing.

A total of 471 *Campylobacter* isolates were collected during 2018–2021 from swine fecal samples or cecal contents from Sichuan Province. The overall prevalence rate of *Campylobacter* was 43.2% (471/1,090), and the prevalence rate varied among the samples derived from different years, with 43.1% (*n* = 144/334) in 2018, 39.6% (*n* = 149/376) in 2019, 44% (*n* = 88/200) in 2020, and 50% (*n* = 90/180) in 2021 (Figure 1A). Before the antibiotic prohibition, the overall prevalence rate of *Campylobacter* in swine feces was 41.3%, and after that, the overall prevalence rate of *Campylobacter* in swine feces had a slight increase and reached 46.8%. Of the *Campylobacter* isolates, 95.5% (450/471) were identified as *C. coli*, and 3.1% (15/471) were determined to be *C. jejuni* by PCR. The remaining six isolates were of different *Campylobacter* spp. than *C. jejuni* and *C. coli* and were not characterized further to the species level.

### Antimicrobial resistance patterns among *Campylobacter* isolates

Overall, *Campylobacter* showed high rates of susceptibility to meropenem and tigecycline (Supplementary Table 2), and erythromycin resistance levels decreased from 92% in 2018 to 62% in 2021. However, the levels of resistance to gentamicin and florfenicol increased significantly between 2018 and 2021; gentamicin resistance increased from 78% in 2018 and 72% in 2019 to 90% in 2020 and 94% in 2021, and the florfenicol resistance rate fluctuated between 14 and 22% from 2018 to 2020 but sharply increased to 72% in 2021. Tetracycline resistance rates remained at a high level and fluctuated between 78 and 84% throughout the study period, while ampicillin resistance remained fairly stable (between 30 and 38%). Both SAPs (Strains isolated after AGP prohibition) and SBPs (Strains isolated before AGP prohibition) showed high rates of resistance to ciprofloxacin (between 96 and 100%) (Figure 1B and Supplementary Table 2).

### Antibiotic resistance genes of *Campylobacter* isolates

**FIGURE 2:**
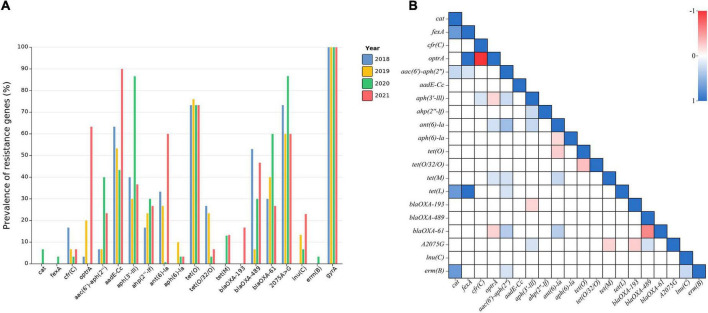
Antibiotic resistance genes in *Campylobacter* isolates. **(A)** The distribution of resistance genes in *Campylobacter* isolates. **(B)** Co-occurrence of ARGs in *Campylobacter*. Significant pairwise values from 1 (blue) to −1 (red) (that is, co-occurrence and mutual exclusivity) are shown.

Based on the genomic analysis, we examined ARGs to tetracycline, aminoglycoside, β-lactam, phenicols, and macrolide, which are important antimicrobial agents in human and/or veterinary medicine. The identified tetracycline resistance genes in *Campylobacter* isolates included *tet*(O), *tet*(M), and *tet*(O/32/O). The prevalence of the *tet*(O) gene remained fairly stable from 2018 to 2021 (∼73.3%), but the average prevalence of the *tet*(O/32/O) gene decreased from 25% in 2018–2019 to 5% in 2020–2021, and the *tet*(M) increased from 0 to 13.3%; 86.7% of the isolates (*n* = 104) harbored at least one *tet* gene. A number of ARGs to aminoglycoside were identified, including *aadE*-Cc, *aac(6′*)-aph(2″), *ant(6)-la*, *aph(3″)-lll*, and *aph(2″)-lf*. Most notably, the prevalence of the *aadE*-Cc gene, which mediates resistance to gentamicin, rose rapidly since the ban of AGPs in feed, from 63.3% in 2018 to 90% in 2021. Although chloramphenicol was banned in food-producing animals in China, florfenicol, a fluorinated thiamphenicol derivative, is still in use. The phenicol resistance genes identified in the sequenced *Campylobacter* isolates included *cat*, *fex*A, *cfr*(C) and *optr*A. Notably, the prevalence of the *optrA* gene, which confers cross-resistance to florfenicol and linezolid, sharply increased from 3.3% in 2018 to 60% in 2021. The prevalence of the *cfr*(C) gene, mediating resistance to five structurally unrelated drugs, fluctuated between 3.3 and 16.7% during the study period. The prevalence of *lnu*(C) has increased over time, from 0% in 2018 and 13.3% in 2019 to 6.7% in 2020 and 23.3% in 2021. The identified β-lactam resistance genes included *bla*_*OXA*–193_, *bla*_*OXA*–489_, and *bla*_*OXA*–61_. The *bla*_*OXA*–193_ gene emerged in *Campylobacter* in 2021, and its percentage was 16.7%. Eighty percent of the isolates (*n* = 96) harbored at least one of these β-lactam resistance genes. Additionally, macrolide and fluoroquinolone resistance were mainly mediated by point mutations in 23S rRNA and *gyr*A, respectively. The prevalence of the 2075A > G mutation remained relatively high (60–86.7%), and all 120 sequenced strains contained the *gyr*A (T86I) mutation (Figure 2A and Supplementary Table 3). These data highly matched the results of antibiotic susceptibility testing.

In addition, several AGRs coexist frequently (Figure 2B), such as *cat* with *tet*(L) and *erm*(B), *tet*(L) with *fex*A, *aac(6′)-aph(2″*) with *fex*A, and *cfr*(C) with *aph(3′-III*). Some ARGs coexisted less in the same strain, such as *optrA* with *aph(3′-III*), *tet*(O) with *aph(6)-la* and *tet*(O/32/O), *bla*_*OXA*–61_ with *bla*_*OXA*–489_, and *bla*_*OXA*–193_ with *aph(3′-III*) (Figure 2B and Supplementary Table 4).

### Multilocus sequence typing and phylogenetic analysis of *Campylobacter coli* isolates of swine and patient origin

**FIGURE 3:**
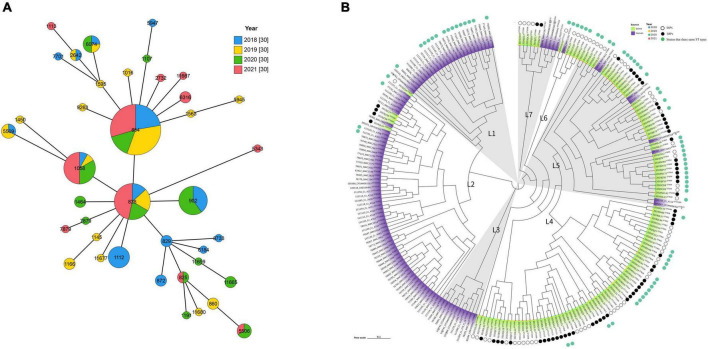
Phylogenetic analysis of *Campylobacter* isolates. **(A)** ST typing of 120 *Campylobacter* isolates over 4 years. Blue: 2018 (*n* = 30), Yellow: 2019 (*n* = 30), Green: 2020 (*n* = 30), and Pink: 2021 (*n* = 30). Numbers in circles represent ST-types. **(B)** SNP phylogenetic tree of 120 swine-derived *Campylobacter* isolates and 105 human-derived *Campylobacter* isolates from a public database. The superscript means ST type; Purple: human-derived; Bright green: swine-derived; Black solid dots: SBPs; white hollow dots: SAPs; blue solid dots: same MLST types shared by swine- and human-derived *Campylobacter* isolates.

Multilocus sequence typing assigned the 120 isolates to 34 distinct sequence types (STs), except for six isolates with novel STs (Supplementary Table 5). The most prevalent STs were ST854, ST828, ST1058, and ST902, with 25 (20.8%), 16 (13.3%), 15 (12.5%), and 10 (8.3%) isolates, respectively. The prevalence of ST1508 and ST828 increased since the ban of AGPs in 2020. Conversely, ST854 and ST1112 were more prevalent before the ban (Figure 3A). Although most isolates showed host specificity and were unique to the isolates’ origin, both MLST and core-genome single nucleotide polymorphism (SNP)-based phylogenetic analysis showed commonality between strains from swine and human, typified by genotypes ST828, ST854, ST825, ST1113, ST1464, ST1595, ST872, and ST1145 (Figure 3B; Litrup et al., 2007).

### Virulence gene profiles of *Campylobacter* isolates

**FIGURE 4:**
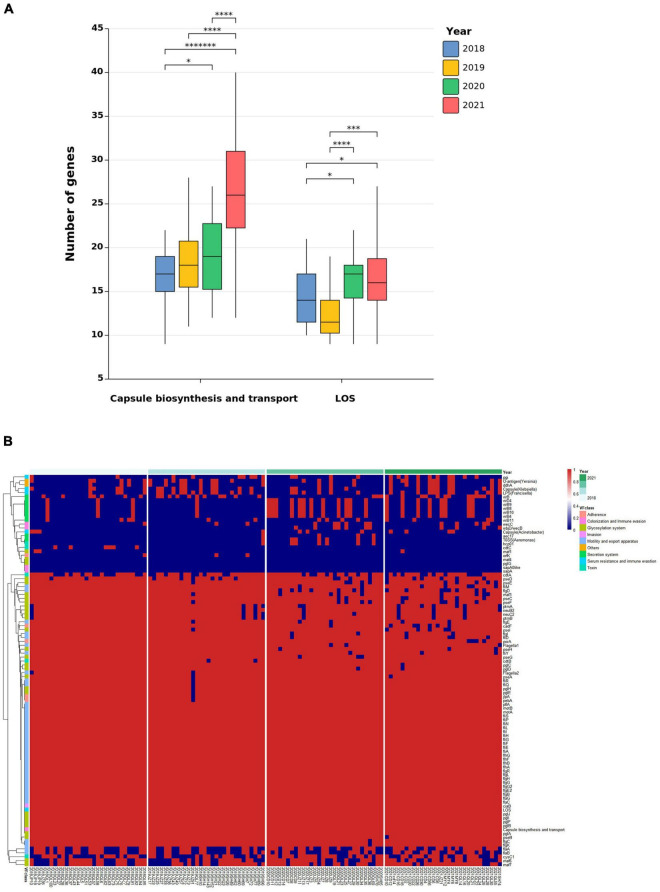
The distribution of virulence genes in *Campylobacter* isolates. **(A)** Predicted numbers of capsule- and LOS-related virulence genes in *Campylobacter* isolates (Blue: 2018, Yellow: 2019, Green: 2020, and Pink: 2021; the median number of genes is denoted as the horizontal line inside the boxplot. *p < 0.05; ^***^p < 0.0001; ^****^p < 0.00001, ^*******^p < 0.0000001). **(B)** Heatmap of *Campylobacter* virulence genes isolated over 4 years (hierarchical clustering of *Y*-axis virulence genes, blue: absence, and red: presence).

*Campylobacter* virulence genes are of great significance for the colonization and adaptation of the strain to the host. A total of 100 virulence genes were clustered into 8 classes and 27 groups. No obvious trends or changes in virulence genes responsible for adherence, glycosylation system, motility and export apparatus, secretion system, or toxin were observed. However, the average number of virulence genes responsible for capsule biosynthesis and transport has increased over time (from 17 in 2018 to 26 in 2021). Additionally, LOS-related genes responsible for serum resistance and immune evasion increased significantly in *Campylobacter* isolates after banning the use of antibiotics as growth promoters (Figure 4A and Supplementary Table 6). These genes mediate the anti-phagocytosis of *Campylobacter* to the host and play an important role in intercellular adhesion and biofilm formation. Further analysis found that SAPs had a higher prevalence than SBPs in genes responsible for immune evasion and secretion systems (T4SS and other related genes). However, O-linked flagellar glycosylation-related genes and flagella-related genes, which are important genes that modify the glycosylation of flagellum and synthesize components of flagella (Song et al., 2015), were absent in some SAPs (Figure 4B).

### Pan-genomic analysis

**FIGURE 5:**
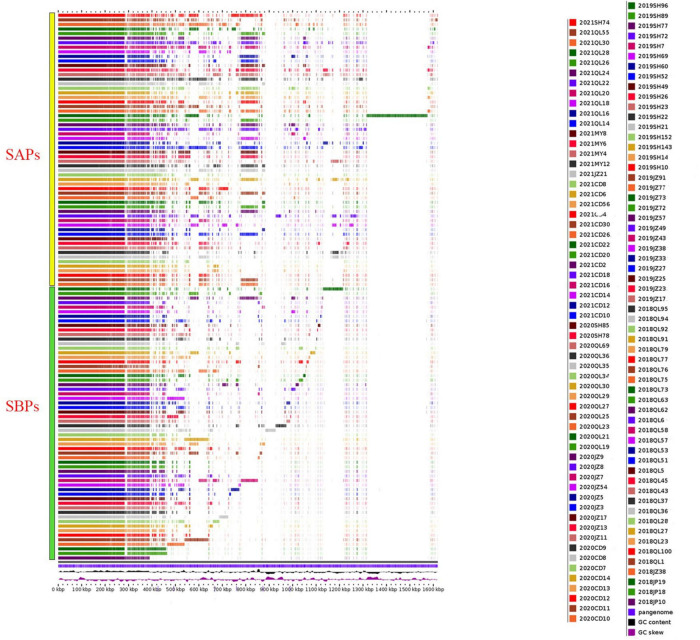
Pan-genomic analysis of 120 *Campylobacter* strains of porcine origin. Each row represents the sequence of one strain. Yellow: SAPs, green: SBPs. Pan-genome, GC content, and GC skew are indicated by blue-purple, black, and purple at the bottom.

The results of the pan-genomic analysis are shown in Figure 5. A clear observation can be made from the figure that, the genomes of SAPs have more accessory genes than SBPs.

## Discussion

Our study explored the influence of withdrawing AGRs from swine diets on the prevalence and antibiotic resistance profile of *Campylobacter* in pigs. After the AGP ban, there was relatively little increase in the isolation rate of *Campylobacter*. However, the prevalence rate of ARGs mediating resistance to gentamycin and florfenicol, as well as the virulence genes responsible for capsule biosynthesis and transport, increased significantly. When comparing the MLST and genomic sequence, commonality exists between *Campylobacter* isolates of swine origin and patients’ origin, indicating potential transmission between swine and humans.

Antibiotic growth promotion in agricultural animal production has been practiced for approximately 50 years. The use of AGPs in feed not only significantly reduces the diversity of the animal’s gut microbiome (Antonopoulos et al., 2009) but also promotes bacterial genome evolution to combat antibiotic pressure (Davies and Davies, 2010). Banning AGPs frees gut microbes from antibiotic stress, and *Campylobacter* adapts to changes in its environment. Although the addition of antibiotics to feed has been eliminated, antibiotics in veterinary medicine, especially in the treatment of infectious diseases, are still in use. At present, the antibiotics commonly used for treatment in Chinese pig farms include: gentamicin, flupenthixol, tilmicosin, doxycycline, enrofloxacin, amoxicillin, amikacin, virginiamycin, etc. The observation that the dissemination of florfenicol resistance in *Campylobacter* dramatically increased after the withdrawal of AGPs might be attributable to the following: (I) although chloramphenicol has been banned, other antimicrobial agents within the same class, such as florfenicol, were widely used as therapeutic drugs in livestock, and provided selection pressure; (II) coselection by other antimicrobial agents might have contributed to the transmission of phenicol-related genes; for example, based on our previous study (Tang et al., 2020), the *cfr*(C) gene was located in a plasmid together with *aph(3′-III*) and *tet*(O). These genes can be coselected when exposed to any one of the corresponding antimicrobial agents; (III) the overuse of disinfectants can also promote the transmission of ARGs when they colocalize with disinfectant resistance genes. In recent years, due to outbreaks of infectious diseases, such as African swine fever, disinfectants have been widely used in swine farms and have placed selection pressure. The prevalence rate of antibiotic resistance genes such as tetracyclines, quinolones and nalidixic acid has always been maintained at a high level. However, it is noteworthy that we observed a decline in the resistance rate of the tested *Campylobacter* to erythromycin, and since macrolide antibiotics are clinically important for the treatment of *Campylobacter* infections, the decline in their resistance rate is a positive feedback for the prohibition of AGPs. It is an interesting phenomenon regarding the presence of *tet*(M) in *Campylobacter*. In the current reports, the resistance of *Campylobacter* to tetracycline is mainly mediated by the *tet*(O), *tet*(O/32/O), and *Cme*ABC genes, and there are few reports in the literature on the presence of *tet*(M) genes in *Campylobacter* and mediating drug resistance (Taylor and Courvalin, 1988; Gibreel et al., 2007). Therefore, further experimental studies are needed to determine whether the *tet*(M) gene found in the *Campylobacter* tested mediates drug resistance. Quinolone resistance caused by mutation of the *gry*A gene exists in all sequenced isolates, indicating that quinolones are no longer against infections caused by *Campylobacter* in this fraction (Hormeño et al., 2016). Both *cfr*(C) and *optr*A mediate cross-resistance to florfenicol and linezolid. However, the phenomenon that the prevalence rate of *cfr*(C) shows a decreasing trend over time, while the prevalence of *optr*A sharply increases in 2021, needs further investigation. Overall, no significant decrease in the antibiotic resistance (except for erythromycin) in *Campylobacter* was observed after the prohibition of antibiotics as growth promoters. Conversely, some antibiotic resistance rates are rising.

Although diverse MLSTs were observed in *Campylobacter* isolates derived from both before and after the AGP ban, the prevalence of some ST types increased or decreased over time, probably because these strains acquire or lose resistance genes or virulence genes that make them better or no longer able to adapt to environmental stress. Both MLST and genomic data showed that some swine- and human-derived strains share high genetic similarity, suggesting that swine-derived *Campylobacter* may be transmitted to humans through pork products, which is in line with previous research (Gorkiewicz et al., 2002; Sheppard et al., 2009).

The capsule biosynthesis and transport system is responsible for bacterial survival and persistence in the environment and evasion of the host immune response (Bacon et al., 2001). LOS diversity is important for the ability to colonize a wide variety of hosts and intestinal niches (Guerry et al., 2002). Their average numbers in SAPs increased significantly, which may indicate that SAPs are stronger than SBPs in terms of persistence in the environment and invasion of organisms. Another obvious difference in the virulence genes before and after the ban of AGPs was that the abundance of secretion system (*vir*B\D)-related genes in the SAP strains increased significantly. The isolates in 2018 and 2019 had fewer *vir*B\D genes, but the genomes of most SAPs contain T4SS-related genes. The basic secretion system function in *Campylobacter* is achieved by flagella (Guerry, 2007); however, some *Campylobacter* acquires type IV (T4SS) or type VI (T6SS) secretion systems. Type IV secretion systems are membrane-associated transporter complexes used by Gram-negative and Gram-positive bacteria to deliver substrate molecules to various target cells in a contact-dependent manner (Alvarez-Martinez and Christie, 2009). T4SS contributes to pathogenesis in multiple ways, including increased genomic plasticity, spread of antibiotic resistance, enhanced surface colonization and biofilm formation, and specific injection of virulence proteins (Kienesberger et al., 2011). The increase in the abundance of the type IV secretion system after the banning of APGs indicated that the virulence of SAPs may be stronger than that of SBPs. The type IV secretion system also mediates the horizontal transfer of bacterial genes, and strains with T4SS have more possibilities for gene export and acquisition (Cascales and Christie, 2003). This is important for the acquisition and dissemination of genes detrimental to humans by the *Campylobacter* genome. Interestingly, we also observed that more O-linked flagellar glycosylation-related genes (*pse*) and Flagella-related genes were absent in SAPs than in SBPs. O-linked flagellar glycosylation and Flagella-related genes play important roles in the function of flagella (Song et al., 2015). Therefore, its absence in turn leads to impaired flagellar function.

Similar to the results of virulence gene prediction, the results of pan-genomic analysis showed that *Campylobacter* strains possess more accessory genes and their genomes are more diverse after the prohibition of AGPs. Combining the trends in virulence genes with the results of the pangenome analysis, we suggest that the possible reason for this is that the prohibition of AGPs produces drastic changes in the environment in which *Campylobacter* lives. The use of AGPs inhibits competition between bacteria by suppressing the growth and reproduction of intestinal bacteria (Lee et al., 2017; Tang et al., 2017). This inhibitory effect is long-term and persistent because AGPs are mixed with the feed. Once AGPs are banned, this inhibitory effect disappears and competition between bacteria for ecological niches increases. The use of therapeutic antibiotics also inhibits the growth of colonies, but its effect is short-term because farms do not generally use large amounts of therapeutic antibiotics continuously. After the ban of AGPs, the fierce competition among colonies will force *Campylobacter* to evolve to adapt to the new survival environment. These evolutions can be characterized by changes in the genome, as in this study with the increase of virulence genes as well as other accessory genes.

## Conclusion

This study represents a systematic investigation of the impact of AGP withdrawal on the foodborne pathogen *Campylobacter*. Samples were collected for four consecutive years, including 2 years before the AGP ban and 2 years after that. We found that the isolation rate of *Campylobacter* slightly increased after the AGP ban, but the florfenicol- and gentamicin-related ARGs increased significantly. Additionally, the number of virulence genes responsible for capsule biosynthesis and the transport system, serum resistance and immune evasion also significantly increased. And the accessory genome was also more enriched. Genomic alignment showed that swine- and human-derived isolates share the same ST types. These data suggested that after antibiotic growth promoters are banned, pathogenic infections may increase, leading to increased use of therapeutic antibiotics on farms and thus to increased rates of antibiotic resistance in *Campylobacter*.

## Data availability statement

The datasets presented in this study can be found in online repositories. The names of the repository/repositories and accession number(s) can be found below: https://www.ncbi.nlm.nih.gov/, PRJNA817390.

## Author contributions

RW: conceptualization, methodology, software, formal analysis, and writing—original draft preparation. CL: methodology and writing—original draft preparation. MZ: data curation. HW and YT: conceptualization, methodology, and funding acquisition. All authors contributed to the article and approved the submitted version.

## Footnotes

1
http://cge.cbs.dtu.dk/services/ResFinder/

2
http://www.mgc.ac.cn/VFs

3
http://cge.cbs.dtu.dk/services/MLST/

4
http://cge.cbs.dtu.dk/services/CSIPhylogeny/

5
https://itol.embl.de/

6
https://rast.nmpdr.org

7
https://server.gview.ca
